# Functional somatic disorders: discussion paper for a new common classification for research and clinical use

**DOI:** 10.1186/s12916-020-1505-4

**Published:** 2020-03-03

**Authors:** Christopher Burton, Per Fink, Peter Henningsen, Bernd Löwe, Winfried Rief, on behalf of the EURONET-SOMA Group

**Affiliations:** 1 Academic Unit of Primary Medical Care, University of Sheffield, Northern General Hospital, Samuel Fox House, Sheffield, S5 7AU UK; 2 Research Clinic for Functional Disorders and Psychosomatics, Aarhus University Hospital, Aarhus, Denmark; 3 Department of Psychosomatic Medicine and Psychotherapy, University Hospital Rechts der Isar, Technical University of Munich, Munich, Germany; 4 Department of Psychosomatic Medicine and Psychotherapy, University Medical Center Hamburg-Eppendorf, Hamburg, Germany; 5 Department of Clinical Psychology and Psychotherapy, University of Marburg, Marburg, Germany

**Keywords:** Classification, Functional disorders, Medically unexplained symptoms, Psychosomatic medicine, Somatoform disorders, Psychophysiologic disorders, Somatic symptom disorder, Bodily distress

## Abstract

**Background:**

Functional somatic symptoms and disorders are common and complex phenomena involving both bodily and brain processes. They pose major challenges across medical specialties. These disorders are common and have significant impacts on patients’ quality of life and healthcare costs.

**Main body:**

We outline five problems pointing to the need for a new classification: (1) developments in understanding aetiological mechanisms; (2) the current division of disorders according to the treating specialist; (3) failure of current classifications to cover the variety of disorders and their severity (for example, patients with symptoms from multiple organs systems); (4) the need to find acceptable categories and labels for patients that promote therapeutic partnership; and (5) the need to develop clinical services and research for people with severe disorders.

We propose ‘functional somatic disorders’ (FSD) as an umbrella term for various conditions characterised by persistent and troublesome physical symptoms. FSDs are diagnosed clinically, on the basis of characteristic symptom patterns. As with all diagnoses, a diagnosis of FSD should be made after considering other possible somatic and mental differential diagnoses. We propose that FSD should occupy a neutral space within disease classifications, favouring neither somatic disease aetiology, nor mental disorder. FSD should be subclassified as (a) multisystem, (b) single system, or (c) single symptom. While additional specifiers may be added to take account of psychological features or co-occurring diseases, neither of these is sufficient or necessary to make the diagnosis. We recommend that FSD criteria are written so as to harmonise with existing syndrome diagnoses. Where currently defined syndromes fall within the FSD spectrum – and also within organ system-specific chapters of a classification – they should be afforded dual parentage (for example, irritable bowel syndrome can belong to both gastrointestinal disorders and FSD).

**Conclusion:**

We propose a new classification, ‘functional somatic disorder’, which is neither purely somatic nor purely mental, but occupies a neutral space between these two historical poles. This classification reflects both emerging aetiological evidence of the complex interactions between brain and body and the need to resolve the historical split between somatic and mental disorders.

## Background

Here we propose a new classification: ‘functional somatic disorder’ (FSD). We apply this term to disorders characterised by certain patterns of physical symptoms, rather than a single consistent cause or pathology. We understand these disorders as having complex aetiological mechanisms, which may vary between individuals with similar symptoms and which are the subject of continuing research. These disorders are common and present in around one-third of healthcare consultations in both primary care [1] and specialist practice [2].

This paper is based on discussions by members of the informal European research network EURONET-SOMA (European Network to Improve Diagnostic, Treatment and Health Care for Patients with Persistent Somatic Symptoms) [3]. The network’s discussions and outputs so far have included research agendas for FSD [4], position statements on outcome measures [5] and aetiological mechanisms [6] and a comparison of healthcare for persistent somatic symptoms across Europe [7, 8]. The purpose of the discussions, which have been conducted since 2016 and are presented in the current paper, was to create a common framework for the classification of FSDs, including the various functional somatic syndromes, for research and clinical use. The authors constitute the core group, but a broader group of people has contributed at some of the face-to-face meetings.

## Rationale for a new classification

We base the argument for a new classification on five factors. To some extent these overlap and some have been rehearsed before [9–12]. Nevertheless, the continued emergence of critiques of current and proposed classifications suggests that problems relating to the classification of FSDs have not yet been adequately resolved. The five factors are:

Developments in understanding the aetiological mechanisms underlying functional symptoms and FSD.

The problem of division of FSDs according to the treating specialist (e.g. irritable bowel syndrome, IBS, in gastroenterology, or somatic symptom disorder in Psychiatry).

Failure of current classifications to cover the variety of disorders and the range of severity within disorders.

The need to find acceptable illness categories and labels for (and with) patients, which promote therapeutic partnership.

The need to develop clinical services and further research for people with severe FSDs.

### Current classifications

At present, two major clinical classification systems involve FSDs: the World Health Organisation’s International Classification of Diseases (ICD) [13] and the American Psychiatric Association’s Diagnostic and Statistical Manual (DSM) [14]. We also consider a recent research classification: bodily distress syndrome (BDS) [15] and proposals for the next version of ICD for primary healthcare (ICD-PHC) [16].

ICD includes all somatic and mental conditions, including – in the versions for primary care – individual or non-specific symptoms. DSM is restricted to the domain of psychiatry and does not have sections for organ or physiological systems (e.g. the gastrointestinal system) in the same way as ICD.

ICD-10 includes individual functional somatic syndromes, such as IBS or fibromyalgia, placed within organ-specific chapters. However, at the same time, so-called ‘medically unexplained symptoms’ – either multiple and across organ systems, or single and related to one organ system – are coded as ‘somatoform’ or ‘dissociative neurological disorders’ within the mental disorders chapter. Their main feature is “repeated presentation of physical symptoms … in spite of repeated negative findings and reassurances by doctors that the symptoms have no physical basis.” This emphasis on “no physical basis” for symptoms is removed in the proposal for ICD-11, which instead introduces bodily distress disorder (BDD) [17]. BDD is “characterized by the presence of bodily symptoms that are distressing to the individual and excessive attention directed toward the symptoms …” . BDD in ICD-11, as well as somatic symptom disorder in DSM-5 [14], focus on the psychological and emotional features relating to physical symptoms as “excessive thoughts, feelings, or behaviours related to the somatic symptoms or associated health concerns”. None of these recent classifications make stipulations about the presence or absence of somatic disease.

In contrast, BDS [15, 18] comprises clusters of symptoms according to organ or physiological system and does not include any requirement for symptoms to be accompanied by psychobehavioural features. It does not include or exclude somatic disease, but states that symptoms should not be better explained by other conditions. In these regards, BDS resembles the specialty-specific functional somatic syndrome diagnoses such as IBS and fibromyalgia, which are also based on physical symptoms reports. The ICD and DSM criteria were originally based on theories about psychological and emotional factors in illness and, later on, consensus based descriptions, with field trials testing the proposals only thereafter. However, the BDS model was, from the start, derived empirically from clinical descriptive and epidemiological studies of physical symptoms. The work underpinning BDS is reflected in proposals for the next version of ICD-PHC. This introduces, within the mental disorders, a category of ‘bodily stress syndrome’ [19]. This proposes a specific number of symptoms not otherwise explained and largely related to a postulated underlying mechanism of autonomic arousal [16].

In parallel with these mental classifications across body systems, several specialty-specific classifications continue to evolve, including criteria for functional gastro-intestinal disorders [20] and fibromyalgia [21].

### Developments in understanding the aetiological mechanisms underlying functional symptoms and functional somatic disorders

There is increasing recognition that FSDs involve multiple processes. While no single aetiological mechanism has been identified for FSDs, studies support the involvement of a variety of processes. Current hypotheses include processes involving both the body (e.g., immune system [22], autonomic nervous system [23], hypothalamo-pituitary axis [24], mitochondrial function [25]) and the brain and mind (processing and perception of bodily signals [6], central sensitisation [26], psychological adaptation [27]). This involvement of multiple processes is thought to be shared across individual syndromes [26, 28], even though the specific processes involved may differ between individuals and syndromes. A common classification may thus strengthen research into aetiological mechanisms in functional disorders and must be capable of further evolution in the light of new scientific knowledge.

### Division of functional somatic disorders according to the most commonly treating specialist

For over 20 years, it has been recognised that there is a rather arbitrary delimitation of single, specialty-specific functional somatic syndromes, such as IBS or fibromyalgia, and that there is great overlap between them in terms of shared symptoms and i patients fulfilling criteria for more than one syndrome [11]. Nevertheless, these remain useful constructs for specialists, generalists and for many patients suffering from symptoms from predominantly one organ or physiological system. There is, thus, a good case that they should be retained and that, where possible, new classifications for FSD should harmonise with these specialty-specific syndromes.

### Failure of any current classifications to include the variety of disorders and the range of severity within disorders

The current separation of syndromes into somatic or mental sections of classifications means that there is no overall category for FSDs. This is a particular problem when patients experience multiple symptoms from multiple organ systems (meeting criteria for more than one functional syndrome or for multiorgan-type BDS) [15], but do not demonstrate the psychological features necessary for a diagnosis in the mental health sections of the ICD-11 or DSM-5.

### The need to find acceptable illness categories and labels for (and with) patients, which promote therapeutic partnership

There is increasing.recognition of the importance of concordant views about illness between patients and their clinicians [29, 30]. Current classifications, which classify some FSDs as somatic and others as mental disorders, cause problems for patients and clinicians. One element of acceptability is the issue of the names used [10, 31, 32]. The term ‘functional’ has clear advantages, although we recognise the unfortunate legacy that it may be used pejoratively by some clinicians [33]. ‘Bodily distress’ is more problematic in (British) English because it implies a lack of mental resilience [10], although that is not the case in several European languages. While the term ‘medically unexplained symptoms’ continues to be used by professionals (especially in primary care), we deprecate its use [10, 34], firstly because it is almost always used about, rather than with, patients. Secondly, it emphasises – rather than addresses – the unhelpful explanatory gap between patients and professionals [35].

### The need to develop services and research into treatments for people with complex functional somatic disorders

Patients with FSDs, particularly those with complex or multisystem FSDs, frequently experience long periods during which conditions are ruled out but no working diagnosis is provided. Having a classification for FSDs that is acceptable across various medical specialties, including primary care, would allow earlier discussion of what a patient has (or might have, when an FSD is still part of a differential diagnosis) rather than waiting for a diagnosis of exclusion, as is currently the case. A similar argument was advanced in relation to the ICD-11 classifications [16].

Such a non-stigmatising and useful diagnostic classification may facilitate an early diagnosis and diminish potential harmful examinations and fruitless symptom treatment approaches. It would also aid the identification of patients for current and potentially future treatments and research.

Additional advantages of this classification are that it could be applied within clinical databases and registries and used to teach students and trainee doctors, where the subject is currently poorly understood [36].

### Purpose of this proposed classification

In view of the five factors discussed above, we aim to classify FSD in a way that will facilitate communication between clinical specialties and bridge the gap between functional somatic syndromes in different sections of the ICD. Furthermore, such a classification will improve representation in epidemiological studies and studies on health care relevance and help to establish corresponding research programs and the provision of syndrome-specific and overarching treatment approaches.

## Key features of the proposed classification

### Functional somatic disorders

We propose the term ‘functional somatic disorders’ as an umbrella term for various conditions characterised by persistent and troublesome physical symptoms that are accompanied by impairment or disability. We understand these symptoms as reflecting the integration of bodily and brain functions and dysfunctions. They are the product of complex interactions, involving multiple biological and psychosocial factors. Diagnosis of FSDs should be made based on the symptoms, not on the presence or absence of specific biological or psychosocial contributors to symptoms.

### Neutral categorical space

FSDs should occupy a neutral space within disease classifications, favouring neither a somatic disease aetiology, nor a mental disorder. This reflects their complex nature and causality and is analogous to pain disorders within ICD-11 [37]. The FSD spectrum includes several established syndromes, such as IBS and fibromyalgia. Where such syndromes fall within the FSD spectrum and also within organ system-specific chapters of a classification, they should be afforded dual parentage (e.g. IBS can belong to both gastrointestinal disorders and FSD). We also recommend that, where possible, FSD criteria are written to harmonise with existing syndrome diagnoses. We have adopted this position to encourage clinicians to think of syndromes as both belonging within their specialist domain and within the broader FSD umbrella, rather than exclusively one or the other. We believe that this degree of flexibility is appropriate in the light of evolving scientific knowledge about both peripheral and central processes underpinning symptoms and disorders.

### Making a diagnosis

The diagnosis of FSDs is essentially clinical. There are no tests that can consistently be used to diagnose the condition(s); instead, diagnosis should be established by a characteristic symptom pattern. As with all diagnoses, it should be made after considering other possible medical and psychiatric differential diagnoses. The patient may, however, have both FSD and another somatic disease or mental or behavioural disorder. For a diagnosis of FSD to be present, symptoms compatible with the diagnosis must have been present for at least 3 months.

### Symptoms within system clusters

Within the FSD classification, physical symptoms are grouped within clusters broadly linked to organ or physiological systems. Currently, we propose six system clusters, derived from clinical descriptive and epidemiological studies, as well as having face validity to many clinicians: musculoskeletal, gastrointestinal, cardiorespiratory, genitourinary, nervous system and fatigue-related. The presence of symptoms across multiple bodily systems is a key element of the proposed classification.

### Relationship to psychological or behavioural features

Patients with FSDs may also have dysfunctional psychological or behavioural features accompanying the bodily symptoms. These features, in themselves, may cause distress and contribute to the symptoms (for instance, through positive feedback loops between symptoms and psychological features); therefore an option is needed to describe these additional treatment needs. Dysfunctional psychological or behavioural features are neither necessary nor sufficient for a diagnosis of FSD, but may be used as an additional specifier within categories.

## Categories of functional somatic disorder

**Fig. 1:**
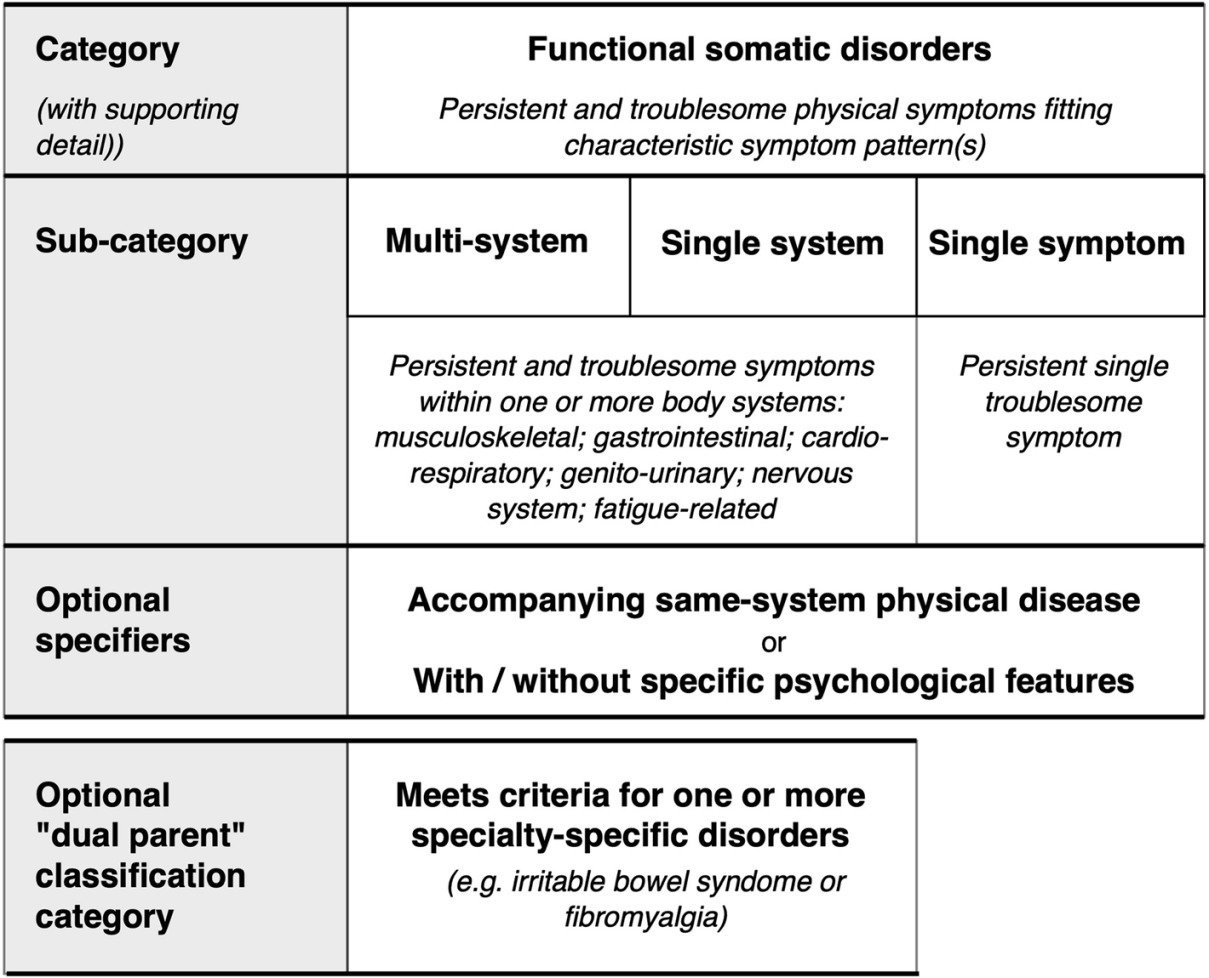
Structure of the proposed classification showing the relationship between main category, sub-categories and additional features

Within the umbrella category of FSD, we propose three categories based on the pattern of physical symptoms and organ or physiological systems involved: (1) multisystem, (b) single system and (c) single symptom. These are summarised in Fig. 1.

### FSD: multisystem type

This is characterised by patients experiencing, at one point in time and over time, multiple persistent and troublesome bodily symptoms across multiple organ or physiological systems that meet further specified classification criteria.

Further work is needed to specify the number of systems and how to grade severity: this may be continuous or categorical, involving both number of symptoms and number of systems. As an example, BDS uses three or more symptoms in three or more body systems [18].

### FSD: single system type

This represents a persistent and troublesome cluster of symptoms that predominantly occur in relation to one bodily system. The current set of symptom clusters is: musculoskeletal, gastrointestinal, cardiorespiratory, genitourinary, nervous system and fatigue-related.

These clusters map closely to some of the existing functional somatic syndromes (such as IBS, fibromyalgia, etc.).

### FSD: single symptom type

This represents an isolated persistent and troublesome symptom (e.g., headache, dizziness, tinnitus). Such single symptoms may or may not, like the other types of FSD referred to above, be associated with dysfunctional psychobehavioural features or symptoms.

### Additional specifiers

In addition to the three categories, we propose two specifiers that may be used to more accurately characterise disorders or to direct treatment:

1. Presence of psychological or behavioural features (cognitive, affective or behavioural) that are dysfunctional; i.e. they cause distress beyond the distress caused by the symptoms themselves; and.

2. Occurrence in interaction with symptom-congruent medical condition (e.g. fibromyalgia in a person with rheumatoid arthritis).

### Relationship of specifiers to diagnosis

The presence or absence of the specifiers is not necessary to diagnose an FSD. In some situations, it may be appropriate to use one or more of them to form more stringent criteria for practice or research.

### Selection of psychological/behavioural characteristics for inclusion as specifiers

Work is in progress to produce a shortlist of characteristics for inclusion. Current work has identified many possible candidates, including health anxiety, catastrophisation, attentional symptom focus, somatosensory amplification, avoidance and safety-seeking behaviour, a general construct of ‘having a weak body’, attributional style, negative affectivity and dissatisfaction with prior health care. The features included in the definition of DSM-5 somatic symptom disorder are not a reliable guidance here. Priority will be given to items that are prognostic in terms of severity/duration or guiding treatment. These features may or may not be part of an additional mental comorbidity.

## Discussion

Our proposed classification of functional somatic disorder is neither purely somatic nor purely mental; it occupies a ‘neutral space’ between these two historical poles. It recognises the complex interplay between body and brain that occurs during the transition from acute to persistent somatic symptoms [6], regardless of whether the symptoms originate in well-defined somatic diseases or arise independently.

Importantly, this proposed diagnosis, based on the symptoms themselves, does not require psychological diagnostic criteria to be present. This is important for clinicians (putting the diagnosis within reach of all specialist areas) and for patients (many of whom are wary about clinicians being ‘too psychological’ too early on in the diagnostic process). By including co-existing psychological symptoms or somatic illnesses, it can be given an extra level of depth of classification.

We believe that the ‘either’ (mental)/‘or’ (somatic) can finally be resolved by this new diagnostic proposal and gives way to a ‘both’, which is more contemporary and scientifically correct. We hope that with it, the unfair and harmful stigmatisations of patients with functional symptoms will diminish and the diagnosis will become more acceptable for people living with FSDs. At the same time, it may help clinicians to remain aware of the high levels of mental comorbidity, in terms of anxiety and depression in people with FSDs [16], which may otherwise remain unrecognised. Notably, our proposal must be further synchronised with other proposals for classification. Just recently, the World Health Assembly adopted the ICD-11 proposal, including the group of chronic pain diagnoses. The ‘chronic primary pain’ category [38] includes many FSDs also mentioned in this manuscript, although the focus of this classification proposal is more on pain as a leading symptom [37]. Both our proposal and the recent pain developments are highly descriptive approaches, avoiding any dualism between psychological versus biological causality. Therefore, our suggested classification of functional somatic syndromes could be located in close proximity to the chronic pain classification and sleeping disorders in ICD-11. However, we see some weaknesses in focusing only on pain diagnosis, even if many other somatic symptoms are present. For instance, diagnosing IBS as a pure pain disorder does not seem to adequately describe this syndrome. ICD-11 offers the opportunity to use double parenting to solve the issue of overlapping categories. Harmonisation of these proposed classifications should be the subject of future work that explicitly encourages the inclusion of different types of symptoms.

Going forward, our diagnostic proposal must prove itself in clinical and scientific practice. We are confident, however, that this proposal is currently the best solution, while remaining open to future scientific developments. We hope that it can be acceptable to patients, scientists and physicians from all fields of medicine and that it will stimulate further research efforts on a national and international level.

## Conclusion

We propose a new classification of functional somatic disorder that is neither purely somatic nor purely mental, but occupies a neutral space between these two historical poles. This reflects both emerging aetiological evidence of the complex interactions between brain and body and the need to resolve the historical split between somatic and mental disorders.

## Data Availability

Not applicable.
